# Transcatheter Aortic Valve Therapy for Bicuspid Aortic Valve Stenosis

**DOI:** 10.3390/jcdd10100421

**Published:** 2023-10-09

**Authors:** Nalin H. Dayawansa, Samer Noaman, Lung En Teng, Nay Min Htun

**Affiliations:** 1 Alfred Health, Melbourne, VIC 3004, Australia; na.dayawansa@alfred.org.au (N.H.D.); s.noaman@alfred.org.au (S.N.); l.teng@alfred.org.au (L.E.T.); 2 Department of Medicine, Central Clinical School, Monash University, Melbourne, VIC 3004, Australia; 3 Baker Heart & Diabetes Institute, Melbourne, VIC 3004, Australia; 4 Western Health, St Albans, VIC 3021, Australia; 5 Peninsula Health, Frankston, VIC 3199, Australia

**Keywords:** bicuspid aortic valve, transcatheter aortic valve implantation (TAVI), transcatheter aortic valve replacement (TAVR), aortic valve stenosis

## Abstract

Transcatheter aortic valve implantation (TAVI) has become first-line treatment for older adults with severe aortic stenosis (AS), however, patients with bicuspid aortic valve (BAV) have been traditionally excluded from randomised trials and guidelines. As familiarity and proficiency of TAVI operators have improved, case-series and observational data have demonstrated the feasibility of successful TAVI in bicuspid aortic valve aortic stenosis (BAV-AS), however, patients with BAV-AS have several distinct characteristics that influence the likelihood of TAVI success. This review aims to summarise the pathophysiology and classification of BAV, published safety data, anatomical challenges and procedural considerations essential for pre-procedural planning, patient selection and procedural success of TAVI in BAV.

## 1. Introduction

Surgical AVR has long been considered the preferred treatment for bicuspid aortic valve aortic stenosis (BAV-AS), however, as transcatheter heart valve (THV) technology has improved and transcatheter aortic valve implantation (TAVI) centres have matured, TAVI has emerged as a safe and feasible treatment for selected patients with BAV-AS. BAV, however, is a distinct clinical entity which poses many challenges to the structural heart team. An understanding of the unique features of BAV is essential during candidate assessment, pre-procedural imaging, THV selection, valve sizing and procedural deployment in TAVI.

## 2. Bicuspid Aortic Valve Disease: Aetiology, Epidemiology and Associated Pathologies

BAV is the most common congenital cardiac anomaly found in adults, affecting 1–2% of the population [1]. Bicuspid valve morphology exists on a continuum, with only 5% of BAV patients exhibiting a true biscuspid phenotype consisting of two symmetrical semi-circular leaflets arising from dual sinuses of Valsalva without evidence of cusp fusion. The majority of BAVs instead exhibit commissural fusion with a fibrous raphe. BAV affects males 2–3 times more commonly than females, and males with BAV have approximately double the incidence of thoracic aortopathy [2].

There is a strong association between BAV and thoracic aortic aneurysm (TAA). Up to 26% of patients with BAV will develop thoracic ascending aorta aneurysms, and patients with BAV have an 8-fold higher risk of thoracic aorta dissection compared to the background population rate [3]. Prevalence of underlying BAV in aortopathy is likely underestimated, with up to 30% of patients with tricuspid aortic valves undergoing ascending aorta repair being found intraoperatively to have partial commissural fusion [4]. BAV also predisposes to coarctation of the aorta, and up to 50% of patients with coarctation will be found to have BAV [5]. Histologically, BAV is associated with diffuse cystic medial necrosis of the ascending aorta: degeneration of the elastic fibres of the intima media with loss of smooth muscle cells [6,7,8]. In addition to aortic wall structural abnormalities, BAV results in abnormal patterns of aortic wall shear stress to eccentric, high-velocity blood flow which further contributes to formation of ascending aortic aneurysm [9].

Several recognised genetic mutations have been observed in association with BAV. Congenital syndromes such as Turner syndrome (45, X0), Down syndrome (trisomy 21), Marfan syndrome and velocardiofacial syndrome (22q11.2 del) are all associated with increased risk of BAV [10]. Loeys–Dietz syndrome, an autosomal dominant connective tissue disorder caused by mutations in TGF-β signalling pathway genes, carries a 10% incidence of BAV and an increased risk of thoracic aortic aneurysm.

Up to a third of patients with non-syndromic BAV exhibit familial clustering in one or more first-degree family members, with an autosomal dominant inheritance pattern and incomplete penetrance [11]. The genetic basis of BAV, however, is heterogenous and polygenic and remains incompletely described. A loss-of-function mutation in *NOTCH1* has been identified in familial clusters of both bicuspid and tricuspid aortic stenosis and is associated with pathological differentiation of pluripotent aortic valve interstitial cells [12,13]. Additional mutations in genes such as *ACTA2*, *TGF-β2*, *FNB01*, *KCNJ2* and *GATA5* have all been associated with BAV and thoracic aortopathy [14]. First-degree relatives of patients with BAV have a 6-fold increase in the risk of BAV and a 3-fold increased risk of aortic dissection [15]. First-degree relatives of BAV probands are recommended to undergo TTE screening for BAV and thoracic aortic aneurysm, however, no clear recommendations have been made for timing of repeat screening despite an increased risk of aortic aneurysm in first-degree relatives with tricuspid aortic valve [16].

All patients with BAV-AS should be screened for ascending aorta aneurysm using both echocardiography and CT aortography as part of routine workup. Currently, guidelines recommend aortic repair where ascending aorta diameter exceeds 5.5 cm or if ascending aorta diameter exceeds 5.0 cm where surgery is being performed in an aorta surgery centre [17]. Aortic repair is indicated in patients with connective tissue disorders or non-syndromic familial TAA where aortic diameter exceeds 5.0 cm or where ascending aorta diameter exceeds 4.5 cm with rapid growth, family history of dissection, or family history of unexplained sudden death. Where aortic valve replacement is indicated for severe BAV-AS, the threshold for concurrent aortic repair is an ascending aorta diameter of more than 4.5 cm [18].

## 3. Bicuspid Aortic Valve Morphology and Classification

Careful planning and preparation are the key to safe and successful TAVI procedures. Similarly, morphological analysis and classification of BAV are key to identifying patients at high risk of complications with BAV-TAVI. Valve morphology is best classified using cardiac CT with image acquisition in both systole and diastole, as sensitivity of echocardiography for diagnosis of BAV may be as low as 11.5% in a TAVI population [19].

**Figure 1:**
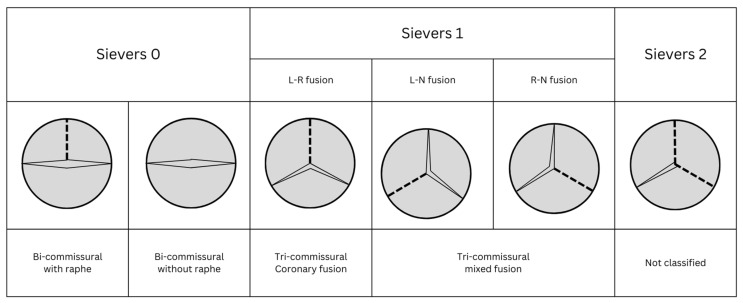
Morphological classification of bicuspid aortic valve. Bicuspid aortic valve (BAV) morphological classification systems as adapted from Sievers (top headings) and Jilahaiwi (bottom headings).

The most widely utilised system proposed by Siever in 2007 classifies BAV based on the number of raphes present and the orientation of the leaflets (Figure 1) [20]. Sievers types 0, 1 or 2 describe the number of raphes present, and the subtype describes the orientation of leaflets (type 0) or leaflet fusion (types 1 and 2). Sievers type 1 L-R, with a single raphe fusing left and right coronary leaflets, is the most common configuration, accounting for 71% of BAVs. Ascending aorta aneurysm is most strongly associated with type 2 BAV, affecting over 50% of patients at the time of AVR.

Jilaihawi et al. proposed an alternate system, with greater consideration of how BAV configuration affects orientation and deployment of TAVI valves [21]. The Jilaihawi system classifies BAV by the number of commissures (counting both fused and functioning commissures) and the presence or absence of a fusion raphe (Figure 1). Sievers type 1 valves may be reclassified as either tricommissural or bicommissural with raphe. Tricommissural BAV describes valves with three symmetrical commissures at 120-degree angles, one of which is partially or completely fused in what is colloquially called ‘functional’ or ‘acquired’ BAV. Bicommissural with raphe-type BAV was observed in younger patients with fusion of asymmetrically sized cusps and a fused raphe oriented perpendicularly to the valve orifice. Fusion orientation was classified as either coronary (LCC-RCC fusion) or mixed (NCC-RCC or NCC-LCC fusion). Sievers type 2 valves cannot be classified within the Jilaihawi system.

Procedurally, coronary cusp fusion is associated with an increased risk of PPM implantation following self-expanding valve implantation [21]. Coronary cusp fusion results in a horizontally oriented valve orifice and may result in a valve expansion being directed anteriorly and posteriorly towards the AV node, rather than laterally. When compared to functional (tricommissural) BAV, congenital (bicommissural with or without raphe) BAV is associated with greater eccentricity of self-expanding TAVI deployment, without a significant difference in expansion ratio [22].

## 4. Clinical Data for Safety and Efficacy of Transcatheter Therapies in Bicuspid AS

Prior to the development of TAVI, treatment of severe AS was limited to surgical valve replacement and balloon valvuloplasty (BV). In pediatric and young adult patients, BV remains a commonly used palliative intervention to either delay AVR or bridge to definitive repair in complex congenital heart defects such as hypoplastic left heart syndrome [23]. BV may result in partial separation of fused commissures, as well as fracture of leaflet calcification; it provides the longest freedom from re-intervention in bicommissural type BAV [24,25]. In adult BAV-AS, the role of standalone BV has diminished as availability and safety of TAVI have risen. Current adult guidelines recommend consideration of standalone BV only as a bridge to TAVI in patients with decompensated heart failure or shock or to facilitate urgent non-cardiac surgery or childbirth [18,26].

Initial feasibility of BAV-TAVI was demonstrated by small case series. Wijesinghe et al. described TAVR in BAV-AS using first-generation balloon-expandable (BE) valves in 11 patients, achieving successful THV deployment in 10 patients [27]. Subsequent larger case series followed, including that of Mylotte et al. which included 139 patients treated with second-generation self-expanding (SE) and BE valves in 2014. Procedural mortality was 3.6%, with a high rate of moderate or severe post-implantation aortic regurgitation in 28.4% of patients. The one-year mortality in this series was 17.5% [28]. There were lower device success rates in the BAV group compared to tricuspid aortic valve (TAV) in this propensity-matched study between 561 BAV patients compared with 4546 TAV patients by Yoon et al. [29].

Third-generation THV prostheses made significant improvements on early designs, implementing a smaller delivery sheath and outer sealing skirts in the SAPIEN-3 (Edwards Lifesciences, Irvine, CA, USA) and a pericardial outer wrap and improved recapture capabilities in the Evolut R/PRO (Medtronic plc, Dublin, Ireland) [30]. Increased use of CT measurements for aortic annulus and root sizing also improved procedural success and reduced the rate of significant para-valvular regurgitation (PVR) [30]. A series of 51 BAV-AS patients treated with SAPIEN-3 by Perlman et al. in 2016 described a high rate of procedural success with no cases of moderate or severe PVR [31]. Data from the Society of Thoracic Surgeon/American College of Cardiology Transcatheter Valve Therapy (STS/ACC TVT) registry later showed no significant difference in rates of mortality and stroke at 30 days following propensity-matched comparison of 932 BAV patients and 26,154 TAV patients treated with self-expanding Evolut R or Evolut PRO valves between July 2015 and September 2018 [32]. Makkar et al. compared 2691 BAV patients treated with SAPIEN-3 with propensity score-matched TAV patients from the STS/ACC TVT registry, also showing no significant difference in the 30-day or 1-year mortality [33]. There were, however, increased rates of pacemaker implantation (9.1% versus 7.5%, *p* = 0.03), stroke (2.5% versus 1.6%, *p* = 0.02) and conversion to open chest surgery (0.9% versus 0.4%, *p* = 0.03) at 30 days.

Over time, data emerged regarding the safety of TAVI in carefully selected BAV-AS patients at low surgical risk. The Low Risk Bicuspid Study published by Forrest et al. was a single-arm prospective study enrolling 150 low-risk patients with BAV-AS with <3% predicted 30-day operative mortality, excluding those with aortopathy, prohibitive LVOT calcification or aged < 60 years old. Following implantation of Evolut or Evolut PRO based on annular measurements, they observed a low rate of all-cause mortality or disabling stroke at 1.3%, with a high device success rate at 95.3% [34]. Contemporary propensity-matched observational studies continue to demonstrate similar outcomes post-TAVI in BAV and TAV patients [35,36]. Meta-analysis comparing BAV-TAVI to TAV-TAVI from separate bicuspid recruitment arms of the PARTNER, Low Risk TAVR and Evolut Low Risk trials showed a statistically significant increase in disabling stroke in BAV-TAVI, however, this was a statistically fragile observation derived from only four events in a cohort of 1421 TAV and 380 BAV patients [37].

THV prostheses have shown comparable longevity compared to bioprosthetic SAVR in tricuspid AS, however, data on THV durability in bicuspid AS are also scarce [38]. Data from the Bicuspid Aortic Valve Anatomy and Relationship with Devices (BAVARD) retrospective registry shows a similar mean gradient between BAV-TAVI and TAV-TAVI at 30 days but significantly reduced THV expansion in BAV-TAVI with reduced indexed effective orifice area (1.17 ± 0.4 cm^2^/m^2^ vs. 1.33 ± 0.37 cm^2^/m^2^, *p* < 0.01) which may compromise THV longevity [39]. The multicenter Italian STABILITY study retrospectively assessed outcomes following BAV-TAVI and observed good haemodynamic valve stability at 4 years post-procedure with only a small increase in mean pressure gradient (13 ± 6.5 mmHg at 4 years vs. 10 ± 5 mmHg at 30 days, *p* = 0.03) [40]. All-cause mortality at four years was 32%, and the incidence of haemodynamic valve failure was 4.1%; both of which are comparable with randomised trial data from tricuspid TAVI in intermediate surgical risk patients [41]. While these data are reassuring, further long-term outcome data are needed to confirm the durability of TAVI in patients with BAV.

Randomised data comparing TAVI to surgical AVR in bicuspid AS are lacking, as BAV patients were excluded from all landmark TAVI RCTs. The largest observational study to date comparing TAVI to SAVR in BAV was a propensity-matched study comparing peri-procedural outcomes with 975 patients in each arm. This study showed similar in-hospital mortality [3.1% vs. 3.1%, OR 1.00, 95% CI 0.59–1.97, *p* > 0.999) and no difference in the rates of cardiac arrest, cardiogenic shock, acute kidney injury, haemopericardium, cardiac tamponade or acute stroke [42]. TAVR was associated with a higher rate of pacemaker implantation (13.8% vs. 4.6%, OR 3.32, 95% CI 2.34–4.71, *p* < 0.01) but a lower rate of bleeding and transfusion. These data suggest short-term safety in a carefully selected cohort of BAV, however, significant details were lacking including type of THV and rate of moderate or greater PVR.

## 5. Anatomical Challenges in TAVI for Bicuspid AS

Individuals with BAV were excluded from randomised controlled trials that examined the effectiveness of TAVI due to a range of structural attributes that might increase the probability of complications during or after the procedure. Notable distinctions persist between individuals with bicuspid and trileaflet AS. Firstly, dimensions of all elements of the aortic valve complex tend to be larger in those with bicuspid morphology compared to their trileaflet counterparts, increasing the likelihood of encountering an annulus size that falls beyond the parameters accommodated by currently available transcatheter heart valves (THVs) [43].

CT and trans-oesophageal echocardiography (TEE) have shown the aortic annulus to have relatively circular geometry in both bicuspid and trileaflet AS, however, supra-annular geometry, particularly at the sinus of Valsalva, tends towards an elliptical form among those with bicuspid anatomy [44,45]. In approximately two-thirds of individuals with BAV, the geometry of the aortic valve (AV) complex, spanning from the annulus to the tips of the leaflets, is flared or tapered rather than tubular [44]. It has been suggested that with this anatomical geometry, the narrowest dimensions and the point of maximum resistance to THV expansion within the AV complex are above the annulus at the level of the commissures [45]. This is especially evident in individuals exhibiting a tricommissural raphe-type anatomy (Figure 1). Elliptical supra-annular geometry may contribute to elliptical deployment, leading to the potential of oversizing THVs and subsequently elevating the risk of insufficient expansion of the THV or damage to the annulus if the sizing is predicated solely on annular dimensions [46]. Data from the BAVARD registry suggest that sizing from the annulus is most appropriate where the AV complex is tubular or flared, however, where the AV complex tapers THV sizing should be taken from the dimensions at the inter-commissural distance 4 mm above the annulus [39].

Stenotic bicuspid valves exhibit a greater degree of calcification in comparison to trileaflet valves. This discrepancy is clearly illustrated by the higher mass observed in histological examination of stenotic BAV [47] as well as the measured calcification on CT [39]. Furthermore, the calcified raphe may indirectly increase the risk of conduction block post-TAVI by intensifying the compression forces exerted on the stent frame adjacent to the non-coronary cusp, which is positioned in close proximity to the His bundle [48].

Human coronary anatomy exhibits a wide spectrum of variability, and patients with BAV are prone to higher take-off of the left main coronary ostium, separate coronary ostia in the left system and increased prevalence of left coronary artery dominance [49,50,51]. While the higher rates of coronary anomalies coupled with the presence of heavily calcified leaflets raises concern for increased risk of coronary obstruction post-TAVI, the height of the coronary ostia tends to be higher in BAV compared to trileaflet AS, mitigating some of this risk [52]. BAV is also associated with increased coronary eccentricity relative to the aortic sinuses, which must be considered during TAVI alignment [53].

BAV patients experience accelerated native valve deterioration and earlier onset of AS compared to trileaflet AV patients, presenting with significant calcific AS most commonly in the fifth or sixth decade of life [54]. Pure, isolated AR is less common and is seen in only 10% of younger BAV patients [55]. Mixed AV disease, combining aortic valve stenosis and regurgitation, intensifies the haemodynamic stress on the left ventricle. Patients with mixed AV disease may be eligible for TAVI, however, data in this subgroup remain limited [56]. Recent studies focusing on BAV patients within the Society of Thoracic Surgeons (STS)/American College of Cardiology (ACC) transcatheter valve therapy (TVT) registry revealed moderate-to-severe AR in 3% to 15% of TAVI-treated patients, however, no specific subgroup analyses assessed outcomes in the mixed AV disease cohort [32,57]. Isolated pure AR cases among BAV patients, typically seen in younger individuals with low calcification burdens and concurrent root aortopathy, are best addressed through surgical replacement or repair, resulting in excellent outcomes [58]. While TAVR might be an option for very high-risk patients with pure AR, its success rate is lower, and comprehensive data on BAV anatomy are limited.

## 6. TAVI Procedural Complications in BAV

### 6.1. Para-Valvular Regurgitation (PVR) and Aortic Root Rupture

Para-valvular regurgitation (PVR) is one of the most common complications of TAVI which occurs due to improper device sealing, and moderate or severe PVR is associated with increased patient mortality [59]. Factors contributing to PVR include stent undersizing, stent eccentricity, high calcium volume and sub-optimal positioning [60]. PVR is one of the most common complications following TAVI in bicuspid AS due to the annular eccentricity and high calcium burden. Oversizing the TAVI prosthesis after careful CT analysis is one approach to reducing the risk of PVR, however, this increases the risk of aortic root rupture during balloon-expandable valve deployment or during balloon post-expansion of self-expanding THVs [61]. The risk of rupture is higher in the presence of substantial calcium volume or a calcified raphe, where protruding calcium may damage native tissue [62,63]. Assessing the risk of aortic root rupture remains challenging due to variations in calcium distribution, as well as patients’ tissue compliance which, while closely linked to age, varies significantly. Reducing balloon filling volume to minimise stress on the native tissue reduces the risk of annular rupture; however, this may elevate the risk of PVR. Balancing the competing risks of PVR and aortic root rupture remains one of the primary challenges during TAVI for patients with complex BAV anatomy. These contrasting risks complicate efforts to avoid moderate-to-severe PVR and aortic root rupture in complex BAV patients during TAVR. BAV patients with considerable calcium volume should be carefully considered for surgical AVR, and a cautious approach should be advised if TAVI is necessary.

### 6.2. Coronary Access and Risk of Obstruction

Coronary access poses a consistent concern when selecting patients for TAVI, particularly in younger patients where the long-term significance of coronary re-access is greater. The risk of coronary obstruction is linked to several factors such as coronary heights and sinus of Valsalva diameters [64]. Among patients with BAV, the sinus of Valsalva diameters tend to be larger, and there is no significant variance in coronary heights compared to those with trileaflet valves [65]. In certain cases of BAV, the location of the coronary ostium may be located closer to the native commissure. Coupled with extensive calcification, this can reduce the distance between the native tissue and the coronary ostium after TAVI.

The risk of coronary obstruction is a major determinant of THV choice, both for the index procedure and any subsequent valve-in-valve TAVI procedures. This risk is notably increased if the first valve used is a self-expanding prosthesis with supra-annular leaflets [66]. In these cases, the stitching of supra-annular bioprosthetic cusps positions them closer to the coronary ostium due to increased deployment heights. When evaluating young BAV patients with low surgical risk, the decision between TAVI and SAVR should encompass assessments of coronary artery height and location.

### 6.3. Transcatheter Heart Valve Selection

Both balloon-expanding valve (BEV) and self-expanding valve (SEV) prostheses have been demonstrated to be safe in BAV-TAVI, and patient anatomical characteristics should be considered during THV selection. Retrospective data in BAV-TAVI have shown BEV to be associated with a small numerical increase in the risk of annular rupture (1.7% vs. 0.0%, *p* = 0.173), whereas SEVs are associated with a significantly increased rate of moderate or severe PVR (10.8% vs. 0.8%, *p* < 0.001) [67]. After propensity score matching, there was a higher mean gradient at 1 year in the BEV population (11.5 ± 4.3 mmHg vs. 8.5 ± 4.2 mmHg, *p* = 0.0019), postulated to be due to the effect of annular eccentricity on THV expansion in BAV.

## 7. Durability and Structural Valve Deterioration

Longevity of the prosthetic valve is a major consideration in younger patients with BAV-AS to incorporate the likelihood and procedural approach to future re-interventions. Valve durability is believed to be determined by the strain exerted on the native leaflets, which in turn is determined by the stent’s deployed geometry and device haemodynamics [68,69]. Post-deployment stent asymmetry is a recurrently observed occurrence in TAVI, encompassing stent ellipticity and tilting relative to the native sinus [39,70]. Stent eccentricity has been demonstrated to create localised regions of elevated Reynolds shear stress and heightened turbulence intensity [71]. Deployment with tilting relative to the native sinus was observed to prolong fluid residence within the sinus opposite to the angle of the stent [72]. All these circumstances may heighten the risk of thrombosis and potentially diminish the endurance of the device.

Of specific concern are anatomically intricate cases of BAV patients, who often exhibit elliptical openings, elevated calcium volumes, and frequently undergo deployments with reduced balloon volumes to mitigate the risks of aortic root damage. These factors render BAV patients more susceptible to uneven deployments and unfavourable haemodynamic outcomes following TAVI, increasing the risk of valve thrombosis and reduced THV longevity. These concerns are yet to be confirmed, however, owing to the paucity of long-term outcome data following BAV-TAVI.

## 8. Selecting Appropriate Candidates for TAVI in BAV-AS

**Figure 2:**
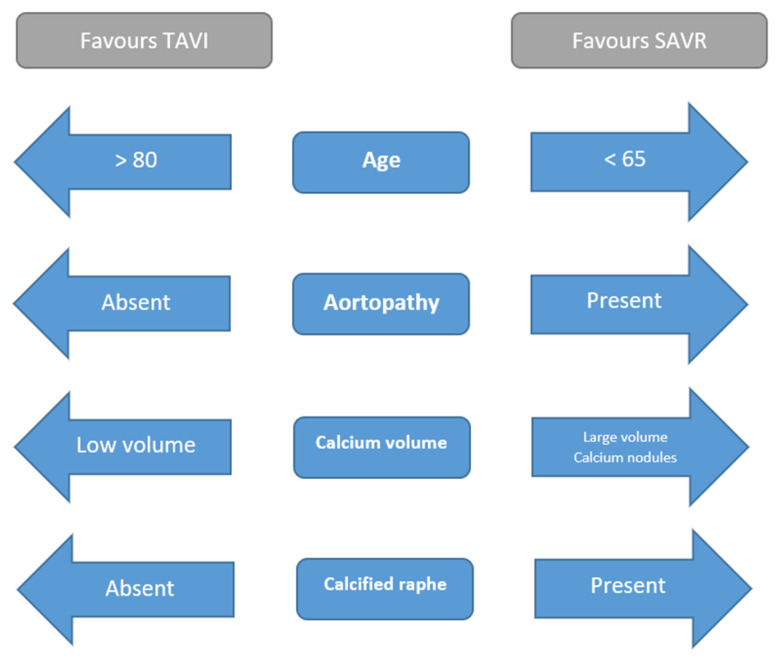
Factors influencing choice of TAVI or SAVR in patients with bicuspid aortic valve.

When selecting patients with BAV-AS appropriate for TAVI, a series of key factors must be considered (summarised in Figure 2). Firstly, the presence of thoracic aortopathy necessitates consideration of surgical valve replacement with concomitant aortic repair. The most recent major guidelines recommend aortic replacement in patients with severe BAV-AS where ascending aorta diameter exceeds 4.5 mm [17]. In patients at extremes of body size, a ratio of ascending aorta area to height of greater than 10 cm^2^/m is considered an indication for ascending aorta repair [17]. Due to the increased eccentricity of ascending aorta geometry in patients with BAV, multiplanar CT imaging should be used to assess aorta dimensions rather than TTE.

Thresholds of age where SAVR is preferred to TAVR in tricuspid AS have lowered considerably as TAVI experience and long-term data have grown. In patients with severe tricuspid AS at low surgical risk, SAVR is preferentially recommended below the age of 75 in European guidelines and below the age of 65 in US guidelines [18,26]. While no formal guidelines exist for selecting patients with BAV for TAVI, a panel of experienced BAV-TAVI operators have recommended heart team discussion for patients with BAV-AS aged between 65 and 80 to weigh the merits of SAVR and TAVR on a case-by-case basis incorporating background life expectancy, predicted surgical risk and technical favourability for TAVI [73]. The best established datasets on safety of BAV-TAVI in younger patients can be drawn from the PARTNER 3 bicuspid registry and the Low Risk Bicuspid Study which enrolled patients with mean age of 71.0 and 70.3, respectively, however, long-term outcomes from these studies are not yet available [34,36].

BAV patients with a high-volume calcium burden should be viewed with caution due to the increased risk of adverse outcomes with TAVI. BAV-AS patients with a calcified raphe and with high volume of leaflet calcification have an increased risk of all-cause mortality following TAVI, with higher rates of aortic root injury and moderate or severe para-valvular leak [74]. Sharma et al. reported a single case experience of successful balloon aortic valve lithotropsy using three parallel peripheral vascular lithotripsy balloons (Shockwave Medical, Santa Clara, CA, USA) to facilitate delivery and expansion of a self-expanding THV in a patient with heavily calcified BAV-AS [75].

## 9. Conclusions and Unanswered Questions

While pioneering operators have demonstrated the feasibility and overall safety of TAVI in patients with BAV, many questions remain unanswered. Randomised trial data comparing TAVI to surgical AVR in bicuspid AS are lacking, however, many ethical, practical and financial barriers exist to conducting such trials. The eccentric annular geometry of BAV-AS is associated with reduced THV expansion, however, longer-term observational data are needed to establish whether reduced THV expansion and higher post-procedure gradients will result in reduced prosthesis longevity. Pre-procedural decision-making pathways of patient selection, THV selection and sizing will continue to be refined and assessed with emphasis on the importance of careful multimodality imaging analysis. With time, the challenge of re-intervention for recurrent AS following BAV-TAVI will increase in frequency, and techniques for valve-in-valve TAVI will need to be adapted to the unique geometry of the BAV valvular complex. There can be no doubt that use of TAVI in BAV will continue to expand, and ongoing detailed observational, imaging and mechanistic study will continue to guide refinements of BAV-TAVI patient selection, planning and technique.

## Data Availability

Not applicable.
